# Ferroptosis in Friedreich’s Ataxia: A Metal-Induced Neurodegenerative Disease

**DOI:** 10.3390/biom10111551

**Published:** 2020-11-13

**Authors:** Piergiorgio La Rosa, Sara Petrillo, Maria Teresa Fiorenza, Enrico Silvio Bertini, Fiorella Piemonte

**Affiliations:** 1 Department of Psychology, Division of Neuroscience, Sapienza University of Rome, 00185 Rome, Italy; piergiorgio.larosa@uniroma1.it (P.L.R.); mariateresa.fiorenza@uniroma1.it (M.T.F.); 2 Unit of Muscular and Neurodegenerative Diseases, Bambino Gesù Children’s Hospital, IRCCS, 00146 Rome, Italy; sara.petrillo@opbg.net (S.P.); enricosilvio.bertini@opbg.net (E.S.B.)

**Keywords:** ferroptosis, Friedreich’s Ataxia, iron, neurodegeneration, oxidative stress

## Abstract

Ferroptosis is an iron-dependent form of regulated cell death, arising from the accumulation of lipid-based reactive oxygen species when glutathione-dependent repair systems are compromised. Lipid peroxidation, mitochondrial impairment and iron dyshomeostasis are the hallmark of ferroptosis, which is emerging as a crucial player in neurodegeneration. This review provides an analysis of the most recent advances in ferroptosis, with a special focus on Friedreich’s Ataxia (FA), the most common autosomal recessive neurodegenerative disease, caused by reduced levels of frataxin, a mitochondrial protein involved in iron–sulfur cluster synthesis and antioxidant defenses. The hypothesis is that the iron-induced oxidative damage accumulates over time in FA, lowering the ferroptosis threshold and leading to neuronal cell death and, at last, to cardiac failure. The use of anti-ferroptosis drugs combined with treatments able to activate the antioxidant response will be of paramount importance in FA therapy, such as in many other neurodegenerative diseases triggered by oxidative stress.

## 1. Introduction

Cell death can be considered as an event in which a cell ceases performing its functions. Although it can be triggered by the inability to rescue overwhelming damages, most of the cell death mechanisms are characterized by active, regulated and evolutionary selected biological processes that, in normal conditions, confer advantages to the organisms. This concept is easily understandable in multicellular organisms, in which cell death represents a fundamental process that determines the targeted elimination of specific cells, potentially dangerous for the whole organism, maintaining tissues homeostasis and regulating specific phases of development [1,2]. Nevertheless, programmed cell death also has been reported in yeast and other unicellular eukaryotes [3,4], as well as in prokaryotes [5]. Two opposing scenarios characterize the regulated processes of cell death [6]: in the first, developmental programs or tissue turnover may determine cell removal in absence of any environmental perturbation. In this context, the programmed death occurring in the vertebrate nervous system takes place, where 50% of the initial neuronal pool undergoes elimination [7], or the NSM sister cell death during embryonic and post embryonic development of *C. Elegans* [8]. On the other hand, mechanisms of cell death can also be triggered by intra- and extra-cellular environmental perturbations that, exceeding the cellular adaptive responses, do not allow the restoration of cellular homeostasis [6].

Historically, cell death has been classified by morphological alterations into three main processes: (i) Type I cell death or apoptosis, defined by shrinkage of the cellular cytoplasm, condensation of chromatin, nuclear fragmentation and formation of membrane delimitated apoptotic bodies; (ii) Type II cell death or autophagy, accompanied by vacuolization of the cytoplasm; (iii) Type III cell death or necrosis, characterized by the rupture of plasma membrane and loss of cellular content [2,6,9]. In recent years, novel signaling pathways triggering cell death have been characterized, and new classifications have been proposed, more focused on molecular aspects and signal transduction mechanisms [6]. Among the plethora of programmed cell death processes recently described, here we focus on ferroptosis, a form of iron-dependent programmed cell death triggered by intracellular redox dysregulation, membrane lipid peroxidation and mitochondrial impairment [10,11].

Before its formal definition, ferroptosis was already documented in vitro by two independent groups, who demonstrated that the erastin-mediated inhibition of the cystine/glutamate antiporter system (XC-), responsible for the Glutathione (GSH) synthesis, or the RAS-selective lethal 3 (RLS3)-mediated inactivation of the GSH peroxidase 4 (GPX4) enzyme, both were able to kill cultured cancer cells leading the oncogenic RAS mutation [12,13]. At the same time, the genetic inhibition of cellular iron uptake or the use of iron chelators were able to prevent this kind of cell death [13,14,15], while an increase in cellular iron content was a positive inducer [13]. Finally, in 2012, ferroptosis was recognized as a form of cell death with discrete morphological, biochemical and genetic features distinguishable from those characterizing apoptosis, necrosis, autophagy and the other forms of regulated cell death [15]. However, the role of ferroptosis is currently still elusive. The most credited hypothesis point to ferroptosis as an adaptive response aimed at eliminating tumorigenic cells, as supported by experiments performed on TP53 mutated cells in which the cystine uptake was inhibited by repressing the expression of SLC7A11 (a XC-system subunit), thus sensitizing cells to this kind of death [16,17,18].

## 2. Ferroptosis: Three Hallmarks for Specific Dysfunctions

The ferroptotic process has been defined as a new type of programmed necrosis triggered by lipid peroxidation and determined by an iron-dependent ROS overload [10,18]. At the morphological level, cells undergoing ferroptosis display a reduction in mitochondrial volume and cristae, and increased mitochondrial membrane density [13,14,15]. Nevertheless, the cell membrane does not break, the nucleus remains in its normal size and no chromatin condensation is observed. The discovery and characterization of ferroptosis inducers has clarified the molecular mechanisms underlying this process. Ferroptosis inducers can be grouped into four classes [10]: (i) those responsible for GSH depletion (the system XC-inhibitors, L-buthionine sulfoximine (BSO), sorafenib, artesunate, erastin and the ferroptosis-inducing agents (FINs) class I (DPI2 and DPI10) [15,19,20,21,22,23,24]. These molecules act by blocking the cystine uptake required for the GSH synthesis, thereby lowering the levels of an essential cofactor for the GPX4 enzyme activity and for the antioxidant system as a whole [12]. (ii) The inducers involved in the direct inhibition of the GPX4 enzyme, whose inactivation causes an uncontrolled increase of lipid peroxides [13]. RLS3 belongs to this class, which interacts with the GPX4 selenocysteine, irreversibly inhibiting the enzyme activity [25], as well as several exponents of the class II FINS (DPI7, DPI10, DPI12, DPI13, DPI17, DPI18 and DPI19) and altretamine, an FDA-approved anti-cancer compound [23,24,26,27]. (iii) The third class of ferroptosis activators (FI56) promotes the degradation of GPX4 and the decrease in Coenzyme Q_10_ cellular levels (CoQ_10_) by inhibiting the mevalonate pathway [28]. (iv) The last group includes the endoperoxide FINO2, which triggers ferroptosis by oxidizing cellular iron and indirectly inhibiting GPX4 activity [29,30]. The use of these molecules has highlighted three essential conditions for starting ferroptosis: the presence of redox-active iron, the increase in lipid peroxidation and the lowering of antioxidant protective defenses [31].

**Figure 1:**
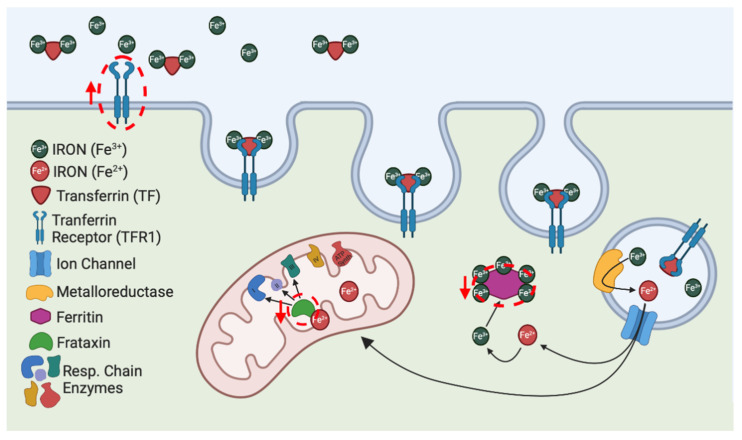
Iron metabolism and its relationship with ferroptosis. Through the transferrin receptor 1 (TFR1), the iron (Fe^3+^) binds to the transferrin carrier (TF) and is imported into the cell by endocytosis. In the endocytic vesicle, Fe^3+^ is reduced to Fe^2+^ and released in the cytosol, where it is targeted to mitochondria for heme (not shown) and iron–sulfur cluster (ISC) synthesis. The intracellular iron excess is stored in ferritin complexes. The deregulation of red-circled proteins (i.e., TFR1, ferritin and frataxin) can trigger ferroptosis.

The iron deregulation plays a critical role in ferroptosis and represents one of the main hallmarks. Iron is a redox-active metal that can contribute to the ROS accumulation via the Fenton reaction. Some enzymes involved in ferroptosis are iron-dependent (e.g., lipoxygenases); thus, the regulation of cellular iron content is critical for preventing ferroptosis. Through the transferrin receptor 1 (TFR1), the iron (Fe^3+^) binds to the transferrin carrier (TF) and is imported into the cell [32]. Inside cells, the TFR1-TF complexes undergo endocytosis, and Fe^2+^ is released in the cytosol [33], where it is targeted to mitochondria for heme and iron–sulfur cluster (ISC) synthesis [34]. The intracellular excess of iron is neutralized by the protein complex ferritin, which consists of 24 light (FTL) and heavy (FTH1) chains subunits [35]. Both increased TRF1 and decreased FTL and FTH1 expression have been found in ferroptosis-sensitive Ras-mutated cancer cells [13], while iron chelators have been shown to inhibit ferroptosis [13,14,15] (Figure 1). The increase in cellular iron content constitutes the major effector of lipid peroxidation, as lipid peroxides are produced by iron-dependent mechanisms [36] and polyunsaturated fatty acids (PUFAs), such as arachidonic acid (AA) and adrenic acid (AdA), being highly susceptible to oxidation [37,38,39]. Notably, only the membrane-incorporated oxidized PUFAs can trigger ferroptosis. Indeed, the genetic ablation and the pharmacological inhibition of enzymes regulating the AA and AdA membrane insertion have been shown to inhibit ferroptosis [39,40,41].

Although in the cellular environment both exogenous (i.e., α-tocopherol) and endogenous (CoQ_10_) molecules may contribute to the detoxification of membrane lipid peroxides, the enzyme GPX4 plays a predominant role by conversion of the membrane hydroperoxides to lipid alcohols [42] using GSH as substrate [19]. The GPX4 relevance in ferroptosis is underlined by the evidence that its ablation during the embryonic development was lethal in mice [43,44], and all the ferroptosis-inducing compounds act, directly or indirectly, on GPX4 expression or activity [15,20,21,22,23,24,25,29,30]. The expression of GPX4 is strictly related to the activity of NRF2, which controls the expression of almost 1% of the genome in the cell, participating to the regulation of multiple cellular processes [45,46,47,48], particularly in the cellular response to oxidative stress and to the maintenance of redox homeostasis [49,50]. NRF2 modulates the expression of many genes implicated in ferroptosis, including GPX4 [51,52]; the genes regulating GSH synthesis (such as GSH synthetase (GSS) [53] and Glutamate-cysteine ligase (GCL) [53,54]; those involved in GSH substrate supplier (the XC-system subunit SLC7A11 [55]); and in GSH recycling (GSH reductase (GSR) [53,54]). NRF2 (NF-E2 p45-related factor 2) also mediates the expression of genes that are critical for iron metabolism (i.e., heme synthesis, iron export and storage) [51,53,56,57], NADPH regeneration [51,53,57] and of the peroxisome proliferator-activated receptor gamma (PPARγ) [58], one of the major regulators of lipid metabolism [59]. Therefore, the transcription factor NRF2 can be considered a key player in the regulation of ferroptosis and its active role has been evidenced also in cancer cells resistant to ferroptosis cues [60,61,62]. In this regard, it has been shown that several tumor suppressors, such as TP53, fumarase or BAP1, induce ferroptosis under specific conditions [11,63], while negative regulators (i.e., NRF2, GPX4 and SLC7A11) are often overexpressed or hyperactivated in tumors [11,31]. This has certainly opened the field for therapeutic opportunities, exploiting ferroptosis inducers to overcome cancer cell resistance [13,14,15].

## 3. Ferroptosis in Neurodegeneration and in Friedreich’s Ataxia

A strong relationship between ferroptosis and neurodegeneration has been reported. Indeed, several neurodegenerative diseases display cellular iron redistribution and its accumulation in specific areas of the central and peripheral nervous system, with a consequent increase of Fenton-mediated lipid peroxidation [10,11]. Elevated iron levels have been found, for instance, in the hippocampus of patients with Alzheimer’s disease (AD) [64] and in the dopaminergic neurons of the substantia nigra in Parkinson’s disease (PD) [65]. Iron accumulation and GSH impairment are key features also in Huntington’s disease (HD) [66].

Iron is essential in the maintenance of brain metabolism, being involved in fundamental processes, such as neurotransmitter and myelin synthesis [67]. On the other hand, iron accumulation is one of the main risk factors in neurodegenerative diseases, as iron accumulation increases with age [68,69] and high iron levels have been found in specific brain regions [64,65,66,70,71].

Mechanisms of cell death different from apoptosis and ascribable to ferroptosis have been detected in several neurodegenerative disorders, including amyotrophic lateral sclerosis (ALS) [72] and PD [73]. In PD, the α-syn dysregulation has been functionally linked to the metabolism of iron and lipids, thus suggesting a possible interplay between ferroptosis and the pathological hallmarks of the disease [74]. Furthermore, anti-ferroptosis molecules were neuroprotective in PD animal models and the iron chelator, deferiprone, slowed disease progression and improved motor function in two independent clinical trials [75,76]. Similarly, in ALS, a dysregulated iron metabolism has been considered as an integral part of the disease pathophysiology [77] and early iron accumulation was identified in neurons of the cortico-spinal motor pathway before neuropathological confirmation [78]. Moreover, markers of DNA oxidation (8-oxo-2′-desoxyguanosine), lipid peroxidation (4-hydroxy-2-nonenal, isoprostane), inflammation (interleukin-6) and iron status (ferritin, hepcidin and transferrin), all closely associated to ferroptosis, have been identified as predictors of disability in 109 patients with ALS, thus contributing to stratify patients for future trials [79].

Among the metal-induced neurodegenerative diseases, Friedreich’s Ataxia (FA) deserves a special focus and this review provides an analysis of the most recent advances linking ferroptosis to the FA pathogenesis (Table 1).

FA is an autosomal recessive trinucleotide expansion disorder, which represents the most common inherited form of ataxia (1:50,000 individuals) [80,81]. In 95% of patients, both alleles display from 200 to 1700 GAA trinucleotide repetitions in the first intron of the FRDA gene, the remaining 5% being caused by a point mutation in the first allele paired with an expanded one [82]. Both conditions determine a decreased expression of the mitochondrial protein frataxin, the FRDA gene product [81], which ranges between 5 and 35% in affected probands, if compared to healthy subjects [82]. The reduction of frataxin expression determines mitochondrial iron accumulation [83], impairments in iron–sulfur cluster (ISC) biogenesis, defective respiratory chain complexes I, II and III and aconitase activities [84], and a reduction in heme biosynthesis [85]; thus, ultimately leading to a Fenton’s reaction-derived ROS overload (particularly superoxide anion and hydroxyl radical-induced lipid peroxides) [86,87,88]. Furthermore, the antioxidant system appears compromised in FA, as a consequence of the defective activity and expression of the transcription factor NRF2, the master regulator of antioxidant cellular responses [89,90,91]. All these defects may predispose cells to ferroptosis in FA, as mounting evidence has recently highlighted [92,93].

Despite it is known since the nineties that frataxin deficiency is the underlying cause of FA [82,94], its function still remains controversial. Most of the proposed frataxin functions are related to iron metabolism and the high degree of sequence conservation among species support a common and evolutionary conserved function in the iron binding [95,96,97]. Given this property, frataxin has been proposed to be an iron storage protein, as also suggested by several studies with purified protein, demonstrating its ability to interact with iron, forming oligomers at a high molecular weight, a function resembling that of ferritin [98,99,100,101]. Frataxin has been shown to participate even in the last step of heme biosynthesis, as supported by its interaction with ferrochelatase, the enzyme responsible for iron incorporation into protoporphyrin IX [85,102,103]. However, the significance of this interaction has to be clarified. Indeed, although a decrease in heme synthesis has been reported in yeast and mouse frataxin mutants [104,105], no alterations were observed in FA erythroid progenitor stem cells, thus suggesting alternative mechanisms able to overcome the frataxin-related defects [106]. Despite this, to date, the frataxin involvement in the ISC biosynthesis remains the most credited function of the protein [107]. Frataxin would directly interact with the complex formed by the cysteine desulfurase NSF1 and the iron–sulfur cluster assembly enzyme ISCU (the major components of the ISC synthesis machinery) [108], although it is not clear whether frataxin may act as iron donor [109] or as an allosteric regulator [110].

In FA, the decreased expression of frataxin determines iron accumulation in tissues, first observed in hearts of patients [111,112] and then in other districts, including the nervous system, especially in the dentate nucleus (DN) of cerebellum and in dorsal root ganglia (DRG) [113,114,115]. Mitochondrial iron overload was confirmed in FA mouse models [105,116,117,118] and cells [119], including FA patients’ fibroblasts [83,120], although technical limits in discriminating between the iron accumulation and/or its redistribution gave rise to contrasting results [116].

Another consequence of the frataxin depletion in FA is the increase in oxidative stress. Early studies demonstrated high susceptibility of FA patient’s fibroblasts to oxidative stress-induced damages [120], and ROS overload was found in most FA animal models, including yeast [121,122], drosophila [123,124,125] and mouse [86,126]. The most credited pathogenic hypothesis in FA is that mitochondrial iron deposits, paralleled by impairments of the mitochondrial ISC-containing enzymes (respiratory chain complexes I-III and aconitase), lead to a Fenton-mediated overproduction of superoxide and hydroxyl radicals [127]. In line with this, elevated levels of oxidative stress markers have been found in FA patients [87,128,129] and extensive lipid peroxidation has been detected in FA cells [130,131,132,133]. Most notably, although one would expect an increased activation of the NRF2-induced antioxidant defenses in FA, as a consequence of oxidative stress and lipid peroxidation, on the contrary the NRF2 signaling pathway appears defective [134] in FA cells [90,135], and its nuclear translocation is compromised [89,90], leading to reduced expression of antioxidant genes [47,90,135], thus exacerbating the vulnerability to oxidative stress.

Iron-induced oxidative stress, together with increased lipid peroxidation and impairment of antioxidant defenses, overall are directed towards ferroptosis as a key mechanism in FA neurodegeneration. Moreover, GSH, an essential cofactor for the GPX4-mediated reduction of lipid peroxidation [19,22], is strongly reduced in blood [136] and fibroblasts [135] of FA patients, accompanied by an increase in protein glutathionylation [137], a process occurring by the spontaneous interaction of oxidized glutathione (GSSG) with protein cysteines [138]. Altogether, these findings further support ferroptosis as a preferential mechanism of cell death in FA. In line with this, fibroblasts derived from skin biopsies of FA patients, from the murine I154F frataxin missense-mutation and from the frataxin Knockin–Knockout (KIKO) mouse model [139], were all found to be hypersensitive to erastin-induced ferroptotic stimuli [92]. Furthermore, while ferroptosis inhibitors were able to rescue the FA cell death determined by ferric ammonium citrate (FAC) iron overload and BSO-dependent GSH depletion, apoptosis inhibitors failed to exert any protective effect [92]. In particular, Du et al. demonstrated that the frataxin silencing lead to mitochondrial dysmorphology in the fibrosarcoma-derived HT-1080 cells, altering membrane potential and respiration and decreasing cell proliferation [93]. As a result, while frataxin knockdown makes HT-1080 cells more susceptible to the ferroptotic process, its overexpression protects HT-1080 cells from erastin-mediated induction of ferroptosis [93]. In addition, analyses of RNA sequencing performed on brown adipose tissue (BAT), obtained from KIKO mice exposed to cold, showed impairment in thermogenesis, concomitant to an increase in ferroptosis markers [140]. A significant decline in NRF2 and GPX4 expression was also evidenced in KIKO embryonic fibroblasts [140], accompanied by an increased adipocyte lethality.

**Figure 2:**
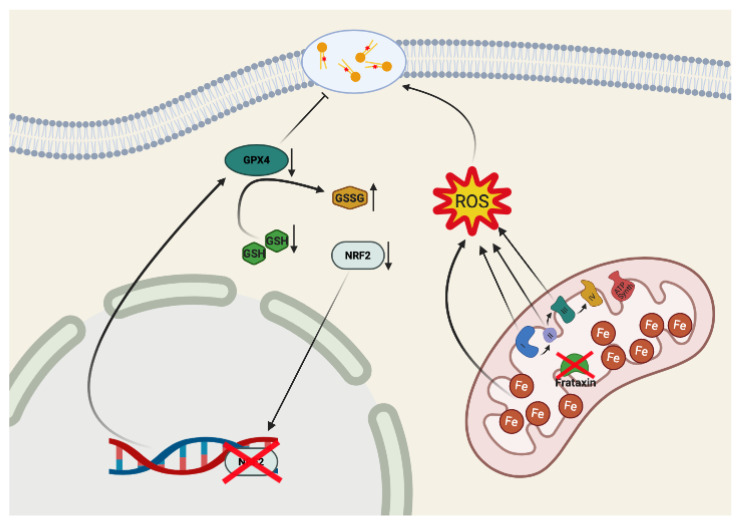
Ferroptosis hallmarks in Friedreich’s Ataxia (FA). In FA, the decrease of frataxin expression determines iron accumulation in the mitochondria and impairments in the Fe–S cluster (ISC) biogenesis, leading to a dysfunctional respiratory chain. The Fenton’s reaction-induced increase of ROS determines the lipid peroxidation of membrane polyunsaturated fatty acids (PUFAs). In FA, the cellular antioxidant defense is faulty, and NF-E2 p45-related factor 2 (NRF2) expression and activity are reduced, leading to a decrease of glutathione (GSH) and GPX4, potentially lowering the threshold needed for ferroptosis induction.

Overall, the evidence strongly identifies FA as a metal-induced neurodegenerative disease and suggest a role for frataxin in maintaining the correct threshold against ferroptosis (Figure 2).

## 4. Conclusions

FA is a neurodegenerative disease in which the degeneration of the DRG neurons and spinocerebellar tracts represent one of the first pathologic events, with cardiomyopathy the main cause of death [80,81]. The frataxin deficiency is the primary cause of the disease [117,141], with the DRG and cerebellum being particularly susceptible to frataxin mutation [126,142]. However, the reason why some cell types (e.g., fibroblasts and peripheral blood mononuclear cells) appear less sensitive to the protein drop is still under investigation [143]. Neurons are highly susceptible to oxidative stress [144], because of the great demand of oxygen [145], high content of transition metals [146] and unsaturated lipids [147], and the relatively low antioxidant defenses [148]. All these aspects are strictly related to ferroptosis, which growing evidence indicates as the main pathogenic mechanism underlying the neurodegeneration in FA. Frataxin depletion would be responsible for lowering the threshold of susceptibility to the ferroptosis induction, thus triggering the neuronal cell death in this disease. Noteworthy, in cancer cells, the frataxin knockdown reduces the growth of xenograft tumors in nude mice. As it occurs in FA, also in these cells the frataxin ablation leads to iron overload, mitochondrial morphology impairment and ISC disassembly, confirming the double role of metal-induced ferroptosis under pathological conditions: potentially beneficial in cancer treatments, but devastating in neurodegenerative diseases [93]. Notably, patients with FA manifest symptoms mostly at the beginning of the second decade of life or later; nevertheless, frataxin deficiency is present from birth, thus suggesting that the metal-induced oxidative damage may accumulate over time, lowering the ferroptosis threshold and leading to neuronal cell death and, at last, to cardiac failure. The use of drugs able to neutralize ROS and lipid peroxidation [47,135] and to activate the NRF2-mediated antioxidant response [91] could be of paramount importance in FA therapy, especially by combining anti-ferroptosis treatments with drugs inducing frataxin expression.

**Table 1:** Ferroptosis-related phenotypes in Friedreich’s Ataxia (FA).

Biochemical Features	Affected Tissues	References
Iron accumulation	Mouse models	[105,116,117,118]
	Cellular models	[84,85,119]
	Human heart	[111,112]
	Human nervous system	[113,114,115]
	Human fibroblasts	[83,120]
Morphological mitochondrial changes	Mouse models	[127]
	Cellular models	[84,93]
ROS and lipid peroxidation	Animal models	[86,88,121,122,123,124,125,126,127]
	Cellular models	[86,92,93,130,131,132,133]
	Human blood	[87,128,129]
	Human fibroblasts	[87,92,120]
Glutathione imbalance	Human blood	[136]
	Human fibroblasts	[87,135,137]
Negative regulation of NRF2	Animal models	[91,140]
	Cellular models	[47,90,91]
	Human fibroblasts	[90,135]
